# 2-(4-Chloro­phen­yl)-4-[1-(4-chloro­phen­yl)-3-methyl-1*H*-pyrazol-5-yl]-5-methyl-1*H*-pyrazol-3(2*H*)-one

**DOI:** 10.1107/S1600536810035713

**Published:** 2010-09-15

**Authors:** Muhammad Rabnawaz, Muhammad Raza Shah, Seik Weng Ng

**Affiliations:** a H.E.J. Research Institute of Chemistry, International Center for Chemical and Biological Sciences, University of Karachi, Karachi 75270, Pakistan; b Department of Chemistry, University of Malaya, 50603 Kuala Lumpur, Malaysia

## Abstract

The title compound, C_20_H_16_Cl_2_N_4_O, has two mol­ecules in the asymmetric unit. The two five-membered rings form a dihedral angle of 54.2 (3)° in one mol­ecule and 56.8 (3)° in the other independent mol­ecule. The amino group of the dihydro­pyrazolone unit of one mol­ecule acts as a hydrogen-bond donor to the carbonyl group of the dihydro­pyrazolone system of the other mol­ecule. The resulting N—H⋯O hydrogen bonds generate a chain running along the *c* axis. The crystal selected was a pseudo-merohedral twin with a 44.9 (3)% twin component.

## Related literature

For the crystal structure of the parent compound without the chlorine-atom substitutents, see: Bertolasi *et al.* (1995 ▶); Kumar *et al.* (1995 ▶).
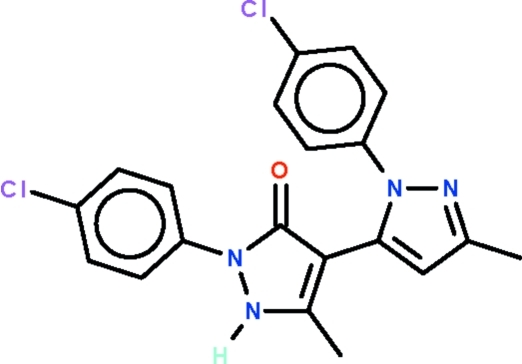


## Experimental

### 

#### Crystal data

C_20_H_16_Cl_2_N_4_O
*M* *_r_* = 399.27
Monoclinic, 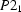

*a* = 7.8095 (2) Å
*b* = 20.2827 (6) Å
*c* = 11.5304 (3) Å
β = 90.075 (2)°
*V* = 1826.39 (9) Å^3^
*Z* = 4
Mo *K*α radiation
μ = 0.37 mm^−1^
*T* = 100 K
0.32 × 0.08 × 0.04 mm

#### Data collection

Bruker SMART APEX diffractometer
Absorption correction: multi-scan (*SADABS*; Sheldrick, 1996 ▶) *T*_min_ = 0.890, *T*_max_ = 0.985
14055 measured reflections
6328 independent reflections
5280 reflections with *I* > 2σ(*I*)
*R*_int_ = 0.090

#### Refinement

*R*[*F* ^2^ > 2σ(*F* ^2^)] = 0.081
*wR*(*F* ^2^) = 0.207
*S* = 1.03
6328 reflections
445 parameters
31 restraints
H-atom parameters constrained
Δρ_max_ = 0.85 e Å^−3^
Δρ_min_ = −0.65 e Å^−3^
Absolute structure: Flack (1983 ▶), 3812 Friedel pairs
Flack parameter: 0.0 (1)

### 

Data collection: *APEX2* (Bruker, 2008 ▶); cell refinement: *SAINT* (Bruker, 2008 ▶); data reduction: *SAINT*; program(s) used to solve structure: *SHELXS97* (Sheldrick, 2008 ▶); program(s) used to refine structure: *SHELXL97* (Sheldrick, 2008 ▶); molecular graphics: *X-SEED* (Barbour, 2001 ▶); software used to prepare material for publication: *publCIF* (Westrip, 2010 ▶).

## Supplementary Material

Crystal structure: contains datablocks global, I. DOI: 10.1107/S1600536810035713/im2224sup1.cif

Structure factors: contains datablocks I. DOI: 10.1107/S1600536810035713/im2224Isup2.hkl

Additional supplementary materials:  crystallographic information; 3D view; checkCIF report

## Figures and Tables

**Table 1:** Hydrogen-bond geometry (Å, °)

*D*—H⋯*A*	*D*—H	H⋯*A*	*D*⋯*A*	*D*—H⋯*A*
N2—H2⋯O2^i^	0.88	1.92	2.65 (1)	139
N6—H6⋯O1	0.88	2.03	2.76 (1)	140

Symmetry code: (i) 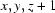.

**Fig. 1.**
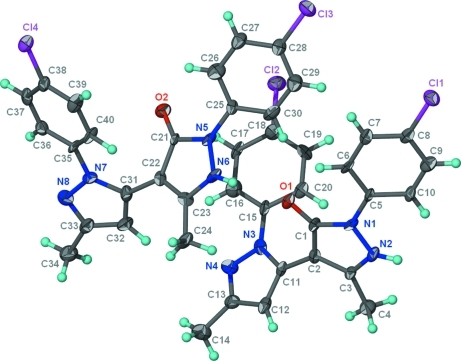
Thermal ellipsoid plot (Barbour, 2001) plot of the two molecules of C20H16ClN4O at the 70% probability level; hydrogen atoms are drawn as spheres of arbitrary radius.

**Fig. 2.**
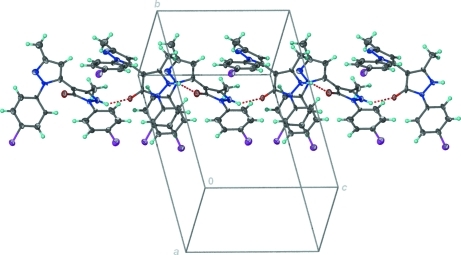
Thermal ellipsoid plot (Barbour, 2001) of the hydrogen-bonded chain structure.

## supplementary crystallographic information

### Comment

We are interested in studying the biological properties of derivatives of 1-phenyl-3-methyl-4(1-phenyl-3-methyl-1*H*-pyrazol-5-yl)-2*H*-3-pyrazolin-5-one. The crystal structure of the parent compound was reported in the context of understanding how amino as well as carbonyl groups connected to a *π*-system influence hydrogen bonding (Bertolasi *et al.*, 1995; Kumar *et al.*, 1995). The chloro-substituted compound (Scheme I, Fig. 1) crystallizes with two independant molecules in the asymmetric unit that display similar bond dimensions. Molecules are linked by N—H···O hydrogen bonds to generate a linear chain running along the *c*-axis of the monoclinic unit cell (Fig. 2).

The crystal studied is a racemic twin; the monoclinic unit cell, having a *β*-angle that is almost a right angle, emulates an orthorhombic unit cell.

### Experimental

1-[1-(4-Chlorophenyl)-3-methyl-5-oxo-4,5-dihydro-1*H*-pyrazol-4-yl]butane-1,3-dione (0.20 g, 0.7 mmol) and 4-chlorophenylhydrazine (0.20 g, 0.14 mmol) were heated in dimethoxyethane and dilute hydrochloric acid for 4 h (synthesis of the dione will be reported elsewhere.). The reaction was quenched by 1 *M* potassium carbonate. The aqueous layer was extracted with ethyl acetate. The combined organic phases were concentrated and the crude product recrystallized from dichloromethane to give 0.19 g of the title compound in 70% yield.

### Refinement

The refinement initially converged at an *R* index of about 20%. As the space group is not a centric space group, the structure was refined as a combination of general and racemic twinning, with the TWIN law of (-1 0 0 0 - 1 0 0 0 1) being used. The refinement on 3812 Friedel pairs gave a Flack parameter of 0.029; the portion of the twin component was 44.9%.

Carbon-bound H-atoms were placed in calculated positions (C—H 0.93 to 0.98 Å) and were included in the refinement in the riding model approximation, with *U*(H) set to 1.2 to 1.5*U*(C). Amino H-atoms were similarly generated.

Some of the diffraction spots of the second and third domain are quite close to those of the main domain affecting the refinement so that the ellipsoids of some atoms are observed to be significantly elongated. The anisotropic temperature factors of five carbon atoms (C1, C3, C10, C21 and C21) were therefore tightly restrained to be nearly isotropic. We have used a very tight restraint (ISOR 0.005). Even with ISOR 0.01, these atoms turn non-positive definite. Phenylene rings were restrained as rigid hexagons with carbon carbon bonds of 1.39 Å each.

A somewhat large WGHT was used that is almost the default value; the *Goodness-of-Fit* was not much different from the default value of 1.

### Crystal data

**Table:**

C_20_H_16_Cl_2_N_4_O	*F*(000) = 824
*M**_r_* = 399.27	*D*_x_ = 1.452 Mg m^−^^3^
Monoclinic, *P*2_1_	Mo *K*α radiation, λ = 0.71073 Å
Hall symbol: P 2yb	Cell parameters from 2188 reflections
*a* = 7.8095 (2) Å	θ = 2.6–22.1°
*b* = 20.2827 (6) Å	µ = 0.37 mm^−^^1^
*c* = 11.5304 (3) Å	*T* = 100 K
β = 90.075 (2)°	Plate, colorless
*V* = 1826.39 (9) Å^3^	0.32 × 0.08 × 0.04 mm
*Z* = 4	

### Data collection

**Table:**

Bruker SMART APEX diffractometer	6328 independent reflections
Radiation source: fine-focus sealed tube	5280 reflections with *I* > 2σ(*I*)
graphite	*R*_int_ = 0.090
ω scans	θ_max_ = 25.0°, θ_min_ = 1.0°
Absorption correction: multi-scan (*SADABS*; Sheldrick, 1996)	*h* = −9→9
*T*_min_ = 0.890, *T*_max_ = 0.985	*k* = −24→24
14055 measured reflections	*l* = −13→13

### Refinement

**Table:**

Refinement on *F*^2^	Secondary atom site location: difference Fourier map
Least-squares matrix: full	Hydrogen site location: inferred from neighbouring sites
*R*[*F*^2^ > 2σ(*F*^2^)] = 0.081	H-atom parameters constrained
*wR*(*F*^2^) = 0.207	*w* = 1/[σ^2^(*F*_o_^2^) + (0.1148*P*)^2^] where *P* = (*F*_o_^2^ + 2*F*_c_^2^)/3
*S* = 1.03	(Δ/σ)_max_ = 0.001
6328 reflections	Δρ_max_ = 0.85 e Å^−^^3^
445 parameters	Δρ_min_ = −0.65 e Å^−^^3^
31 restraints	Absolute structure: Flack (1983), 3812 Friedel pairs
Primary atom site location: structure-invariant direct methods	Flack parameter: 0.0 (1)

### Fractional atomic coordinates and isotropic or equivalent isotropic displacement parameters (Å^2^)

**Table:**

	*x*	*y*	*z*	*U*_iso_*/*U*_eq_	
Cl1	0.7639 (3)	0.50000 (12)	1.1115 (2)	0.0254 (5)	
Cl2	0.2433 (3)	0.73300 (12)	0.5009 (2)	0.0276 (5)	
Cl3	0.6738 (3)	0.50272 (11)	0.6071 (2)	0.0279 (5)	
Cl4	1.3049 (3)	0.73174 (12)	−0.0046 (2)	0.0236 (5)	
O1	0.8167 (9)	0.8010 (3)	0.8422 (5)	0.0189 (14)	
O2	0.7406 (9)	0.8012 (3)	0.3352 (6)	0.0244 (16)	
N1	0.7364 (10)	0.7877 (4)	1.0350 (6)	0.0149 (16)	
N2	0.6547 (9)	0.8275 (4)	1.1178 (7)	0.0196 (16)	
H2	0.6287	0.8163	1.1893	0.023*	
N3	0.6031 (10)	0.9429 (4)	0.7631 (7)	0.0177 (16)	
N4	0.6322 (10)	1.0009 (4)	0.7076 (7)	0.0225 (17)	
N5	0.8150 (10)	0.7850 (4)	0.5275 (6)	0.0166 (17)	
N6	0.8991 (10)	0.8225 (4)	0.6125 (6)	0.0188 (16)	
H6	0.9250	0.8089	0.6829	0.023*	
N7	0.9652 (10)	0.9418 (3)	0.2675 (7)	0.0172 (16)	
N8	0.9481 (10)	1.0025 (4)	0.2169 (7)	0.0218 (17)	
C1	0.7499 (13)	0.8238 (5)	0.9318 (8)	0.021 (2)	
C2	0.6771 (12)	0.8870 (5)	0.9542 (8)	0.0174 (19)	
C3	0.6235 (12)	0.8862 (4)	1.0671 (8)	0.0157 (19)	
C4	0.5388 (13)	0.9395 (4)	1.1409 (8)	0.024 (2)	
H4A	0.4354	0.9215	1.1772	0.036*	
H4B	0.5079	0.9770	1.0916	0.036*	
H4C	0.6188	0.9540	1.2012	0.036*	
C5	0.7512 (8)	0.7199 (2)	1.0537 (5)	0.0164 (19)	
C6	0.8415 (7)	0.6801 (3)	0.9770 (4)	0.018 (2)	
H6A	0.8992	0.6993	0.9128	0.022*	
C7	0.8476 (7)	0.6124 (2)	0.9944 (4)	0.0188 (19)	
H7	0.9093	0.5852	0.9420	0.023*	
C8	0.7633 (8)	0.5844 (2)	1.0884 (5)	0.020 (2)	
C9	0.6729 (7)	0.6242 (3)	1.1650 (4)	0.022 (2)	
H9	0.6153	0.6051	1.2292	0.026*	
C10	0.6669 (7)	0.6920 (2)	1.1476 (4)	0.0184 (19)	
H10	0.6051	0.7192	1.2000	0.022*	
C11	0.6723 (11)	0.9425 (4)	0.8734 (8)	0.0156 (19)	
C12	0.7453 (12)	1.0042 (5)	0.8902 (8)	0.021 (2)	
H12	0.8011	1.0203	0.9577	0.026*	
C13	0.7176 (13)	1.0376 (4)	0.7838 (8)	0.020 (2)	
C14	0.7813 (15)	1.1062 (5)	0.7499 (9)	0.031 (3)	
H14A	0.7385	1.1174	0.6725	0.047*	
H14B	0.9068	1.1065	0.7495	0.047*	
H14C	0.7395	1.1386	0.8061	0.047*	
C15	0.5111 (7)	0.8935 (2)	0.7027 (5)	0.019 (2)	
C16	0.5119 (7)	0.8944 (3)	0.5821 (5)	0.025 (2)	
H16	0.5709	0.9284	0.5420	0.030*	
C17	0.4262 (8)	0.8457 (3)	0.5203 (4)	0.020 (2)	
H17	0.4268	0.8463	0.4379	0.024*	
C18	0.3398 (8)	0.7959 (3)	0.5790 (5)	0.022 (2)	
C19	0.3391 (8)	0.7950 (2)	0.6995 (5)	0.022 (2)	
H19	0.2800	0.7610	0.7396	0.027*	
C20	0.4247 (8)	0.8437 (3)	0.7614 (4)	0.020 (2)	
H20	0.4242	0.8431	0.8437	0.024*	
C21	0.8081 (12)	0.8223 (4)	0.4247 (7)	0.0133 (18)	
C22	0.8897 (13)	0.8838 (4)	0.4551 (8)	0.020 (2)	
C23	0.9332 (12)	0.8821 (4)	0.5691 (8)	0.017 (2)	
C24	1.0216 (13)	0.9322 (4)	0.6447 (8)	0.023 (2)	
H24A	0.9802	0.9280	0.7245	0.034*	
H24B	0.9967	0.9766	0.6158	0.034*	
H24C	1.1455	0.9246	0.6429	0.034*	
C25	0.7768 (8)	0.7182 (2)	0.5441 (5)	0.0152 (19)	
C26	0.8020 (8)	0.6725 (3)	0.4558 (4)	0.028 (2)	
H26	0.8412	0.6868	0.3819	0.034*	
C27	0.7700 (9)	0.6061 (2)	0.4755 (4)	0.025 (2)	
H27	0.7872	0.5749	0.4152	0.030*	
C28	0.7126 (9)	0.5852 (2)	0.5836 (5)	0.019 (2)	
C29	0.6874 (9)	0.6308 (3)	0.6718 (4)	0.024 (2)	
H29	0.6482	0.6166	0.7457	0.029*	
C30	0.7194 (8)	0.6973 (3)	0.6521 (4)	0.024 (2)	
H30	0.7022	0.7285	0.7124	0.029*	
C31	0.8965 (12)	0.9420 (5)	0.3771 (8)	0.019 (2)	
C32	0.8358 (13)	1.0035 (5)	0.3962 (8)	0.024 (2)	
H32	0.7826	1.0189	0.4651	0.029*	
C33	0.8663 (12)	1.0403 (5)	0.2950 (8)	0.023 (2)	
C34	0.8278 (15)	1.1108 (5)	0.2670 (9)	0.029 (2)	
H34A	0.8228	1.1165	0.1827	0.044*	
H34B	0.9181	1.1391	0.2991	0.044*	
H34C	0.7174	1.1231	0.3011	0.044*	
C35	1.0548 (7)	0.8927 (2)	0.2047 (4)	0.0170 (19)	
C36	1.0520 (7)	0.8956 (2)	0.0843 (5)	0.020 (2)	
H36	0.9962	0.9312	0.0462	0.024*	
C37	1.1310 (8)	0.8465 (3)	0.0195 (3)	0.021 (2)	
H37	1.1290	0.8484	−0.0628	0.026*	
C38	1.2127 (7)	0.7945 (3)	0.0752 (4)	0.0174 (19)	
C39	1.2155 (8)	0.7916 (2)	0.1957 (5)	0.026 (2)	
H39	1.2714	0.7560	0.2337	0.031*	
C40	1.1366 (9)	0.8407 (3)	0.2604 (3)	0.021 (2)	
H40	1.1385	0.8387	0.3427	0.025*	

### Atomic displacement parameters (Å^2^)

**Table:**

	*U*^11^	*U*^22^	*U*^33^	*U*^12^	*U*^13^	*U*^23^
Cl1	0.0274 (13)	0.0186 (11)	0.0301 (14)	−0.0004 (12)	0.0030 (11)	0.0032 (11)
Cl2	0.0312 (13)	0.0280 (12)	0.0238 (12)	−0.0023 (13)	−0.0073 (12)	−0.0023 (12)
Cl3	0.0309 (14)	0.0219 (12)	0.0307 (13)	−0.0064 (13)	−0.0026 (11)	0.0031 (11)
Cl4	0.0220 (12)	0.0288 (11)	0.0202 (11)	0.0040 (12)	0.0030 (11)	−0.0016 (10)
O1	0.021 (3)	0.026 (3)	0.010 (3)	0.002 (3)	0.000 (3)	0.001 (3)
O2	0.033 (4)	0.024 (4)	0.016 (3)	−0.002 (3)	0.001 (3)	−0.003 (3)
N1	0.016 (4)	0.019 (4)	0.010 (4)	0.006 (3)	−0.002 (3)	−0.004 (3)
N2	0.018 (4)	0.027 (4)	0.014 (4)	0.001 (3)	0.006 (3)	−0.005 (3)
N3	0.010 (4)	0.021 (4)	0.022 (4)	0.002 (3)	0.005 (3)	0.001 (4)
N4	0.023 (4)	0.019 (4)	0.025 (4)	0.001 (4)	−0.008 (3)	0.001 (4)
N5	0.021 (4)	0.020 (4)	0.008 (4)	0.007 (3)	−0.004 (3)	−0.003 (3)
N6	0.022 (4)	0.029 (4)	0.006 (4)	0.001 (3)	−0.006 (3)	−0.004 (3)
N7	0.012 (4)	0.018 (4)	0.022 (4)	0.002 (3)	0.001 (3)	−0.007 (3)
N8	0.017 (4)	0.021 (4)	0.027 (4)	−0.001 (4)	−0.001 (3)	0.001 (4)
C1	0.028 (4)	0.023 (4)	0.013 (4)	−0.005 (3)	−0.003 (3)	0.002 (3)
C2	0.011 (5)	0.026 (5)	0.015 (4)	−0.004 (4)	−0.002 (4)	0.002 (4)
C3	0.013 (4)	0.019 (3)	0.015 (4)	0.000 (3)	0.007 (3)	−0.003 (3)
C4	0.022 (5)	0.024 (5)	0.025 (5)	0.002 (4)	0.004 (4)	−0.002 (4)
C5	0.014 (4)	0.027 (5)	0.008 (4)	−0.003 (4)	0.001 (3)	−0.003 (4)
C6	0.017 (5)	0.028 (5)	0.010 (5)	0.003 (4)	−0.003 (4)	−0.006 (4)
C7	0.011 (4)	0.031 (5)	0.014 (4)	−0.001 (4)	0.001 (4)	0.001 (4)
C8	0.005 (4)	0.036 (5)	0.018 (5)	−0.002 (4)	0.000 (4)	−0.009 (4)
C9	0.017 (5)	0.028 (5)	0.021 (5)	0.005 (4)	0.000 (4)	−0.005 (4)
C10	0.013 (3)	0.028 (4)	0.014 (4)	−0.002 (3)	0.005 (3)	−0.001 (3)
C11	0.014 (5)	0.023 (5)	0.010 (4)	0.001 (4)	0.003 (4)	0.001 (4)
C12	0.023 (5)	0.026 (5)	0.015 (5)	0.009 (5)	0.008 (4)	0.003 (4)
C13	0.027 (5)	0.015 (4)	0.019 (5)	0.000 (4)	0.001 (4)	−0.003 (4)
C14	0.040 (7)	0.023 (5)	0.031 (6)	−0.001 (4)	−0.009 (5)	0.001 (4)
C15	0.030 (6)	0.015 (4)	0.013 (4)	0.010 (4)	0.009 (4)	0.006 (4)
C16	0.023 (5)	0.030 (5)	0.021 (5)	0.002 (4)	−0.005 (4)	0.008 (5)
C17	0.018 (5)	0.034 (5)	0.007 (4)	0.006 (4)	0.003 (4)	0.001 (4)
C18	0.022 (5)	0.028 (5)	0.015 (5)	−0.002 (4)	−0.005 (4)	0.003 (4)
C19	0.020 (5)	0.019 (4)	0.027 (5)	−0.004 (4)	−0.002 (4)	0.005 (4)
C20	0.013 (5)	0.032 (5)	0.015 (5)	0.003 (4)	0.001 (4)	−0.002 (4)
C21	0.017 (4)	0.015 (3)	0.008 (3)	0.002 (3)	0.004 (3)	0.000 (3)
C22	0.025 (6)	0.011 (4)	0.023 (5)	0.008 (4)	0.009 (4)	0.004 (4)
C23	0.019 (5)	0.010 (4)	0.023 (5)	0.006 (4)	−0.004 (4)	0.006 (4)
C24	0.020 (5)	0.038 (6)	0.011 (5)	−0.003 (4)	−0.002 (4)	−0.005 (4)
C25	0.018 (4)	0.017 (4)	0.011 (3)	0.005 (3)	−0.001 (3)	0.005 (3)
C26	0.031 (6)	0.026 (6)	0.028 (6)	0.008 (4)	0.000 (5)	−0.004 (4)
C27	0.028 (5)	0.025 (5)	0.022 (5)	0.006 (4)	0.005 (4)	0.001 (4)
C28	0.017 (5)	0.025 (5)	0.017 (5)	0.004 (4)	−0.002 (4)	0.003 (4)
C29	0.028 (5)	0.024 (5)	0.021 (5)	0.003 (4)	0.002 (4)	0.000 (4)
C30	0.016 (5)	0.039 (6)	0.017 (5)	−0.001 (4)	0.004 (4)	−0.012 (4)
C31	0.019 (5)	0.027 (5)	0.012 (5)	−0.004 (4)	−0.004 (4)	0.005 (4)
C32	0.032 (5)	0.024 (5)	0.015 (5)	0.007 (5)	−0.002 (4)	−0.004 (4)
C33	0.017 (5)	0.033 (5)	0.019 (5)	−0.008 (4)	0.003 (4)	−0.009 (4)
C34	0.038 (6)	0.022 (5)	0.027 (6)	0.005 (5)	0.000 (5)	0.001 (4)
C35	0.015 (5)	0.024 (5)	0.012 (4)	0.000 (4)	0.008 (4)	−0.005 (4)
C36	0.020 (5)	0.022 (5)	0.018 (5)	−0.006 (4)	−0.001 (4)	0.003 (4)
C37	0.015 (5)	0.031 (5)	0.018 (5)	−0.003 (4)	0.006 (4)	0.002 (4)
C38	0.010 (4)	0.027 (5)	0.015 (5)	−0.001 (4)	0.003 (3)	0.000 (4)
C39	0.027 (6)	0.032 (6)	0.018 (5)	0.006 (5)	0.000 (4)	0.004 (4)
C40	0.030 (6)	0.026 (5)	0.008 (4)	0.011 (4)	0.001 (4)	0.003 (4)

### Geometric parameters (Å, °)

**Table:**

Cl1—C8	1.732 (5)	C14—H14B	0.9800
Cl2—C18	1.734 (5)	C14—H14C	0.9800
Cl3—C28	1.722 (5)	C15—C16	1.3900
Cl4—C38	1.728 (4)	C15—C20	1.3900
O1—C1	1.247 (11)	C16—C17	1.3900
O2—C21	1.234 (10)	C16—H16	0.9500
N1—C5	1.395 (9)	C17—C18	1.3900
N1—C1	1.401 (12)	C17—H17	0.9500
N1—N2	1.405 (10)	C18—C19	1.3900
N2—C3	1.348 (12)	C19—C20	1.3900
N2—H2	0.8800	C19—H19	0.9500
N3—N4	1.358 (11)	C20—H20	0.9500
N3—C11	1.381 (12)	C21—C22	1.445 (13)
N3—C15	1.417 (8)	C22—C23	1.358 (12)
N4—C13	1.330 (12)	C22—C31	1.485 (12)
N5—C25	1.402 (9)	C23—C24	1.506 (12)
N5—N6	1.403 (10)	C24—H24A	0.9800
N5—C21	1.407 (11)	C24—H24B	0.9800
N6—C23	1.337 (11)	C24—H24C	0.9800
N6—H6	0.8800	C25—C26	1.3900
N7—N8	1.368 (11)	C25—C30	1.3900
N7—C31	1.373 (12)	C26—C27	1.3900
N7—C35	1.416 (8)	C26—H26	0.9500
N8—C33	1.345 (12)	C27—C28	1.3900
C1—C2	1.426 (14)	C27—H27	0.9500
C2—C3	1.367 (12)	C28—C29	1.3900
C2—C11	1.462 (13)	C29—C30	1.3900
C3—C4	1.527 (12)	C29—H29	0.9500
C4—H4A	0.9800	C30—H30	0.9500
C4—H4B	0.9800	C31—C32	1.352 (14)
C4—H4C	0.9800	C32—C33	1.406 (14)
C5—C6	1.3900	C32—H32	0.9500
C5—C10	1.3900	C33—C34	1.496 (13)
C6—C7	1.3900	C34—H34A	0.9800
C6—H6A	0.9500	C34—H34B	0.9800
C7—C8	1.3900	C34—H34C	0.9800
C7—H7	0.9500	C35—C36	1.3900
C8—C9	1.3900	C35—C40	1.3900
C9—C10	1.3900	C36—C37	1.3900
C9—H9	0.9500	C36—H36	0.9500
C10—H10	0.9500	C37—C38	1.3900
C11—C12	1.387 (15)	C37—H37	0.9500
C12—C13	1.418 (13)	C38—C39	1.3900
C12—H12	0.9500	C39—C40	1.3900
C13—C14	1.527 (13)	C39—H39	0.9500
C14—H14A	0.9800	C40—H40	0.9500
			
C5—N1—C1	129.8 (7)	C16—C17—H17	120.0
C5—N1—N2	119.9 (7)	C17—C18—C19	120.0
C1—N1—N2	108.1 (7)	C17—C18—Cl2	119.5 (3)
C3—N2—N1	107.2 (7)	C19—C18—Cl2	120.4 (3)
C3—N2—H2	126.4	C18—C19—C20	120.0
N1—N2—H2	126.4	C18—C19—H19	120.0
N4—N3—C11	111.9 (8)	C20—C19—H19	120.0
N4—N3—C15	117.8 (7)	C19—C20—C15	120.0
C11—N3—C15	130.3 (7)	C19—C20—H20	120.0
C13—N4—N3	104.9 (7)	C15—C20—H20	120.0
C25—N5—N6	121.9 (6)	O2—C21—N5	122.3 (8)
C25—N5—C21	128.8 (6)	O2—C21—C22	133.6 (8)
N6—N5—C21	108.4 (7)	N5—C21—C22	104.1 (7)
C23—N6—N5	108.7 (7)	C23—C22—C21	108.8 (8)
C23—N6—H6	125.6	C23—C22—C31	126.7 (9)
N5—N6—H6	125.6	C21—C22—C31	123.8 (8)
N8—N7—C31	110.6 (7)	N6—C23—C22	109.7 (8)
N8—N7—C35	117.5 (7)	N6—C23—C24	119.0 (8)
C31—N7—C35	131.8 (7)	C22—C23—C24	131.1 (9)
C33—N8—N7	105.9 (8)	C23—C24—H24A	109.5
O1—C1—N1	122.8 (8)	C23—C24—H24B	109.5
O1—C1—C2	130.6 (9)	H24A—C24—H24B	109.5
N1—C1—C2	106.6 (8)	C23—C24—H24C	109.5
C3—C2—C1	106.5 (8)	H24A—C24—H24C	109.5
C3—C2—C11	127.5 (9)	H24B—C24—H24C	109.5
C1—C2—C11	125.9 (8)	C26—C25—C30	120.0
N2—C3—C2	111.6 (8)	C26—C25—N5	120.9 (5)
N2—C3—C4	117.5 (8)	C30—C25—N5	119.0 (4)
C2—C3—C4	130.9 (9)	C25—C26—C27	120.0
C3—C4—H4A	109.5	C25—C26—H26	120.0
C3—C4—H4B	109.5	C27—C26—H26	120.0
H4A—C4—H4B	109.5	C28—C27—C26	120.0
C3—C4—H4C	109.5	C28—C27—H27	120.0
H4A—C4—H4C	109.5	C26—C27—H27	120.0
H4B—C4—H4C	109.5	C29—C28—C27	120.0
C6—C5—C10	120.0	C29—C28—Cl3	120.4 (3)
C6—C5—N1	121.0 (4)	C27—C28—Cl3	119.6 (3)
C10—C5—N1	118.9 (4)	C28—C29—C30	120.0
C5—C6—C7	120.0	C28—C29—H29	120.0
C5—C6—H6A	120.0	C30—C29—H29	120.0
C7—C6—H6A	120.0	C29—C30—C25	120.0
C6—C7—C8	120.0	C29—C30—H30	120.0
C6—C7—H7	120.0	C25—C30—H30	120.0
C8—C7—H7	120.0	C32—C31—N7	106.9 (8)
C7—C8—C9	120.0	C32—C31—C22	128.4 (9)
C7—C8—Cl1	121.5 (3)	N7—C31—C22	124.7 (8)
C9—C8—Cl1	118.5 (3)	C31—C32—C33	107.2 (9)
C10—C9—C8	120.0	C31—C32—H32	126.4
C10—C9—H9	120.0	C33—C32—H32	126.4
C8—C9—H9	120.0	N8—C33—C32	109.5 (9)
C9—C10—C5	120.0	N8—C33—C34	119.8 (9)
C9—C10—H10	120.0	C32—C33—C34	130.7 (9)
C5—C10—H10	120.0	C33—C34—H34A	109.5
N3—C11—C12	106.5 (8)	C33—C34—H34B	109.5
N3—C11—C2	126.9 (8)	H34A—C34—H34B	109.5
C12—C11—C2	126.5 (8)	C33—C34—H34C	109.5
C11—C12—C13	104.4 (8)	H34A—C34—H34C	109.5
C11—C12—H12	127.8	H34B—C34—H34C	109.5
C13—C12—H12	127.8	C36—C35—C40	120.0
N4—C13—C12	112.3 (8)	C36—C35—N7	118.3 (5)
N4—C13—C14	120.3 (8)	C40—C35—N7	121.6 (5)
C12—C13—C14	127.4 (9)	C35—C36—C37	120.0
C13—C14—H14A	109.5	C35—C36—H36	120.0
C13—C14—H14B	109.5	C37—C36—H36	120.0
H14A—C14—H14B	109.5	C38—C37—C36	120.0
C13—C14—H14C	109.5	C38—C37—H37	120.0
H14A—C14—H14C	109.5	C36—C37—H37	120.0
H14B—C14—H14C	109.5	C37—C38—C39	120.0
C16—C15—C20	120.0	C37—C38—Cl4	120.3 (3)
C16—C15—N3	118.6 (5)	C39—C38—Cl4	119.7 (3)
C20—C15—N3	121.4 (5)	C38—C39—C40	120.0
C15—C16—C17	120.0	C38—C39—H39	120.0
C15—C16—H16	120.0	C40—C39—H39	120.0
C17—C16—H16	120.0	C39—C40—C35	120.0
C18—C17—C16	120.0	C39—C40—H40	120.0
C18—C17—H17	120.0	C35—C40—H40	120.0
			
C5—N1—N2—C3	−166.1 (7)	Cl2—C18—C19—C20	176.5 (5)
C1—N1—N2—C3	−1.3 (9)	C18—C19—C20—C15	0.0
C11—N3—N4—C13	−1.0 (10)	C16—C15—C20—C19	0.0
C15—N3—N4—C13	179.3 (7)	N3—C15—C20—C19	−178.4 (6)
C25—N5—N6—C23	174.0 (8)	C25—N5—C21—O2	11.0 (15)
C21—N5—N6—C23	4.1 (10)	N6—N5—C21—O2	−180.0 (8)
C31—N7—N8—C33	−0.8 (10)	C25—N5—C21—C22	−170.0 (8)
C35—N7—N8—C33	−177.4 (7)	N6—N5—C21—C22	−1.0 (10)
C5—N1—C1—O1	−16.9 (15)	O2—C21—C22—C23	176.4 (10)
N2—N1—C1—O1	−179.7 (8)	N5—C21—C22—C23	−2.3 (11)
C5—N1—C1—C2	163.7 (8)	O2—C21—C22—C31	5.4 (17)
N2—N1—C1—C2	0.9 (10)	N5—C21—C22—C31	−173.3 (8)
O1—C1—C2—C3	−179.5 (10)	N5—N6—C23—C22	−5.7 (10)
N1—C1—C2—C3	−0.2 (10)	N5—N6—C23—C24	179.2 (8)
O1—C1—C2—C11	−3.1 (17)	C21—C22—C23—N6	5.0 (11)
N1—C1—C2—C11	176.1 (8)	C31—C22—C23—N6	175.7 (9)
N1—N2—C3—C2	1.2 (10)	C21—C22—C23—C24	179.4 (9)
N1—N2—C3—C4	−178.4 (7)	C31—C22—C23—C24	−9.9 (17)
C1—C2—C3—N2	−0.6 (11)	N6—N5—C25—C26	−137.2 (6)
C11—C2—C3—N2	−176.9 (8)	C21—N5—C25—C26	30.5 (11)
C1—C2—C3—C4	178.9 (9)	N6—N5—C25—C30	40.2 (10)
C11—C2—C3—C4	2.6 (16)	C21—N5—C25—C30	−152.1 (7)
C1—N1—C5—C6	23.4 (11)	C30—C25—C26—C27	0.0
N2—N1—C5—C6	−175.6 (5)	N5—C25—C26—C27	177.3 (7)
C1—N1—C5—C10	−153.6 (8)	C25—C26—C27—C28	0.0
N2—N1—C5—C10	7.5 (8)	C26—C27—C28—C29	0.0
C10—C5—C6—C7	0.0	C26—C27—C28—Cl3	−179.9 (5)
N1—C5—C6—C7	−176.9 (6)	C27—C28—C29—C30	0.0
C5—C6—C7—C8	0.0	Cl3—C28—C29—C30	179.9 (5)
C6—C7—C8—C9	0.0	C28—C29—C30—C25	0.0
C6—C7—C8—Cl1	178.7 (5)	C26—C25—C30—C29	0.0
C7—C8—C9—C10	0.0	N5—C25—C30—C29	−177.4 (6)
Cl1—C8—C9—C10	−178.7 (4)	N8—N7—C31—C32	−0.2 (10)
C8—C9—C10—C5	0.0	C35—N7—C31—C32	175.7 (8)
C6—C5—C10—C9	0.0	N8—N7—C31—C22	178.5 (9)
N1—C5—C10—C9	177.0 (6)	C35—N7—C31—C22	−5.6 (15)
N4—N3—C11—C12	1.7 (10)	C23—C22—C31—C32	−48.2 (16)
C15—N3—C11—C12	−178.6 (8)	C21—C22—C31—C32	121.1 (11)
N4—N3—C11—C2	−175.0 (8)	C23—C22—C31—N7	133.4 (10)
C15—N3—C11—C2	4.6 (14)	C21—C22—C31—N7	−57.3 (14)
C3—C2—C11—N3	−128.0 (11)	N7—C31—C32—C33	1.1 (10)
C1—C2—C11—N3	56.4 (14)	C22—C31—C32—C33	−177.5 (10)
C3—C2—C11—C12	55.9 (14)	N7—N8—C33—C32	1.5 (10)
C1—C2—C11—C12	−119.7 (11)	N7—N8—C33—C34	179.6 (9)
N3—C11—C12—C13	−1.6 (9)	C31—C32—C33—N8	−1.6 (11)
C2—C11—C12—C13	175.2 (9)	C31—C32—C33—C34	−179.4 (10)
N3—N4—C13—C12	−0.1 (10)	N8—N7—C35—C36	−28.8 (9)
N3—N4—C13—C14	177.0 (9)	C31—N7—C35—C36	155.5 (8)
C11—C12—C13—N4	1.1 (11)	N8—N7—C35—C40	154.5 (6)
C11—C12—C13—C14	−175.7 (10)	C31—N7—C35—C40	−21.1 (11)
N4—N3—C15—C16	24.3 (9)	C40—C35—C36—C37	0.0
C11—N3—C15—C16	−155.4 (8)	N7—C35—C36—C37	−176.7 (6)
N4—N3—C15—C20	−157.3 (6)	C35—C36—C37—C38	0.0
C11—N3—C15—C20	23.1 (11)	C36—C37—C38—C39	0.0
C20—C15—C16—C17	0.0	C36—C37—C38—Cl4	177.8 (5)
N3—C15—C16—C17	178.5 (6)	C37—C38—C39—C40	0.0
C15—C16—C17—C18	0.0	Cl4—C38—C39—C40	−177.8 (5)
C16—C17—C18—C19	0.0	C38—C39—C40—C35	0.0
C16—C17—C18—Cl2	−176.6 (5)	C36—C35—C40—C39	0.0
C17—C18—C19—C20	0.0	N7—C35—C40—C39	176.6 (6)

### Hydrogen-bond geometry (Å, °)

**Table:**

*D*—H···*A*	*D*—H	H···*A*	*D*···*A*	*D*—H···*A*
N2—H2···O2^i^	0.88	1.92	2.65 (1)	139
N6—H6···O1	0.88	2.03	2.76 (1)	140

Symmetry codes: (i) *x*, *y*, *z*+1.
